# The Dynamics of OXA-23 β-Lactamase from *Acinetobacter baumannii*

**DOI:** 10.3390/ijms242417527

**Published:** 2023-12-15

**Authors:** Roberto Arrigoni, Andrea Ballini, Luigi Santacroce, Luigi Leonardo Palese

**Affiliations:** 1 CNR Institute of Biomembranes, Bioenergetics and Molecular Biotechnologies (IBIOM), 70126 Bari, Italy; r.arrigoni@ibiom.cnr.it; 2 Department of Clinical and Experimental Medicine, University of Foggia, 71122 Foggia, Italy; 3 Interdisciplinary Department of Medicine (DIM), University of Bari ‘Aldo Moro’, 70124 Bari, Italy; luigi.santacroce@uniba.it; 4 Department of Translational Biomedicine and Neurosciences—(DiBraiN), University of Bari ‘Aldo Moro’, 70124 Bari, Italy

**Keywords:** protein structure, molecular dynamics simulation, β-lactamases, drug resistance, microbial, antibiotics, anti-bacterial agents, bacterial proteins, *Acinetobacter baumannii*, carbapenem-resistant Enterobacteriaceae, humans

## Abstract

Antibiotic resistance is a pressing topic, which also affects β-lactam antibiotic molecules. Until a few years ago, it was considered no more than an interesting species from an academic point of view, *Acinetobacter baumanii* is today one of the most serious threats to public health, so much so that it has been declared one of the species for which the search for new antibiotics, or new ways to avoid its resistance, is an absolute priority according to WHO. Although there are several molecular mechanisms that are responsible for the extreme resistance of *A. baumanii* to antibiotics, a class D β-lactamase is the main cause for the clinical concern of this bacterial species. In this work, we analyzed the *A. baumanii* OXA-23 protein via molecular dynamics. The results obtained show that this protein is able to assume different conformations, especially in some regions around the active site. Part of the OXA-23 protein has considerable conformational motility, while the rest is less mobile. The importance of these observations for understanding the functioning mechanism of the enzyme as well as for designing new effective molecules for the treatment of *A. baumanii* is discussed.

## 1. Introduction

**Figure 1:**
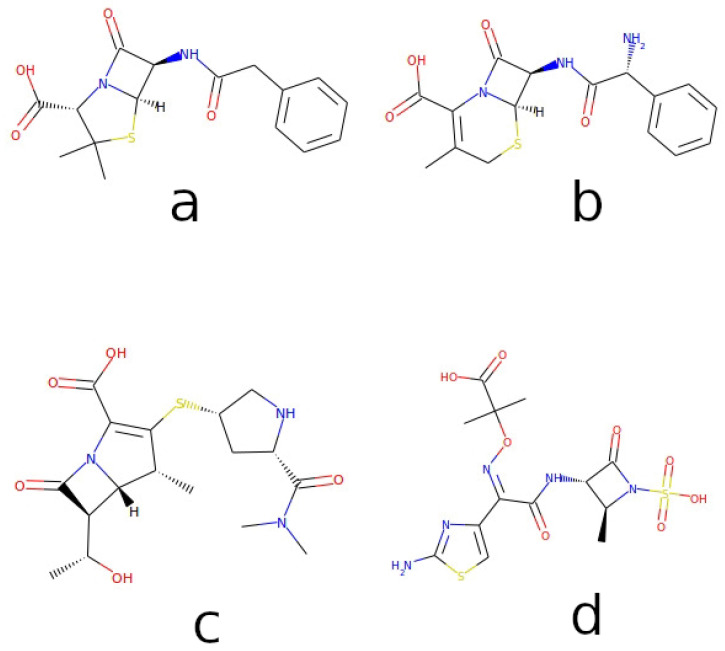
Structures of representative β-lactams. Reported molecules are (**a**) penicillin G or benzylpenicillin, a classic penicillin; (**b**) cephalexin, a first-generation cephalosporin; (**c**) the carbapenem antibiotic meropenem; and (**d**) the monocyclic β-lactam antibiotic aztreonam. Color code: carbon in black, oxygen-containing groups in red, nitrogen-containing groups in blue, sulfur-containing groups in yellow.

The discovery of penicillin [1], whose centenary will soon occur, represented a milestone in the history of medicine: its introduction into clinical practice has literally revolutionized the treatment of microbial diseases, so much so that we can speak of a pre- and post-antibiotic era. This molecule was the progenitor of the class of β-lactam antibiotics that, despite being in use for decades, still represent the most prescribed antibiotic class. β-lactams have been the subject of intense research and development work to improve their potency, spectrum of antibacterial activity, pharmacokinetic profile, and toxicity. Four classes of β-lactam antibiotics are currently used in the clinic: (i) the penicillins, whose four-membered β-lactam ring is fused to a thiazolidine ring; (ii) the cephalosporins, in which the four-membered β-lactam ring is fused to a six-membered dihydrothiazine; (iii) the carbapenems, containing a five-membered pyrroline as a fusion ring; and (iv) the monocyclic β-lactam antibiotics, belonging to the monobactams. Figure 1 reports some representative molecules of these classes.

Unfortunately, a few years after antibiotic discovery, already at the end of 1950s, the first phenomena of resistance began to be reported [2]. Antibiotic resistance, initially associated with enteric bacteria such as *Salmonella*, *Escherichia coli*, and *Shigella*, was initially considered of modest clinical importance because it was limited to a small group of pathogens. That the problem was actually much more serious began to become evident in the 1970s, when strains of *Neisseria gonorrhoeae* and *Haemophilus influenzae* resistant to ampicillin and strains of *Haemophilus* also resistant to tetracyclines and chloramphenicol began to be reported [3]. Since then, the emergence of antibiotic-resistant strains has increased at an impressive rate, up to the recognition of ESKAPE (*Enterococcus faecium*, *Staphylococcus aureus*, *Klebsiella pneumoniae*, *Acinetobacter baumannii*, *Pseudomonas aeruginosa*, and *Enterobacter species*) as a problem for human health that cannot be postponed, for which there is an extreme urgency of new molecules capable of inhibiting their growth [4,5,6]. In addition, antimicrobial resistant strains of *Mycobacterium tuberculosis* and *Mycoplasmataceae* also appeared in the last decades [7,8]. The emergence and spread of antimicrobial resistance, besides the sad load of over 700,000 deaths annually, has a substantial economic cost due to longer medical treatments, increased mortality, and reduced work capacity, which is cumulatively estimated to be 100 trillion USD by 2050, with a concomitant projection to 2050 of 10 million deaths if the current trend is not reversed [9,10,11].

*Acinetobacter* is a genus of Gram-negative bacteria belonging to Gammaproteobacteria found in virtually all environments and is considered as a low-virulence, opportunistic bacteria group of negligible significance until the mid-1990s. Subsequently, epidemiological studies have demonstrated its frequent involvement in hospital infections [12,13]. Clinical manifestations of *Acinetobacter* nosocomial infection are pneumonia and lower respiratory tract infections, urinary infections, wound and burn infections, skin and soft tissue infections, and also as necrotizing fasciitis, meningitis, osteomyelitis, endocarditis, and bloodstream infections. Between these, *A. baumannii* is of major concern due to its ability to acquire quickly antimicrobial resistance traits [14]. In particular, the acquisition of resistance to antibiotics of last resort belonging to the carbapenem class has dramatically increased among *A. baumannii* strains. Currently, multidrug resistant (MDR, resistance to at least three classes of antimicrobials), extensively drug resistant (XDR, MDR plus carbapenem resistance), and pan drug resistant (PDR, XDR plus polymyxin resistance) *A. baumannii* strains are isolated, often rendering currently available treatments ineffective. The situation is so dramatic that *A. baumannii* appears in the list of pathogens for which the research and development of new molecules with antibiotic activity is critical (priority 1, according to the World Health Organization) [15]. Among the numerous resistance mechanisms observed in *A. baumannii* isolates, the one that allowed this pathogen to make a quantum leap as a threat to public health was the acquisition of resistance to carbapenems, mainly, though not exclusively, due to the presence of enzymes capable of inactivating these antibiotics [14,16].

Resistance to β-lactams can occur via several mechanisms: target modifications, such as mutation or alternative forms of the Penicillin Binding Proteins (PBPs); reduction in porin levels, which are proteins necessary for the entry of these molecules into bacterial cells; and the expression of modifying enzymes or efflux pumps [17] can all be involved, even in combination, to explain the phenomenon. But, the ability to produce enzymes capable of inactivating β-lactams is by far the most important mechanism, particularly from a clinical point of view [16,18]. Indeed the ability to produce β-lactamases, enzymes discovered even before the introduction of penicillin in the clinical practice, is crucial for the resistance of a particular bacterial strain to these molecules. Initially discovered in Gram-negatives, enzymes capable of inactivating penicillin were also identified in Gram-positives a few years later [19,20]. Thousands of enzymes of this type are currently known [21], which can be classified in different ways [22,23]. The Ambler system, which is based on sequences, considers four classes, namely A, B, C, and D. Classes A, C, and D contain a serine at the active site (serine β-lactamases), whilst class B comprises different proteins that are zinc metalloenzymes (metallo β-lactamases). Serine β-lactamases employ the serine residue as nucleophile and hydrolyze β-lactams via a covalent acylenzyme intermediate [16].

Class D β-lactamases were referred to as OXAs (oxacillinases) since they are more efficient at hydrolyzing the isoxazolylpenicillin oxacillin than benzylpenicillin [22,24,25]. Some of the subgroups of these enzymes possess carbapenemase activity: OXA-23, OXA-24/40, OXA-48, OXA-51, OXA-58, OXA-134a, OXA-143, OXA-211, OXA-213, OXA-214, OXA-229, and OXA-235. OXA-23, OXA-24/40, and OXA-48 are of particular concern because they are responsible for carbapenem resistance in *A. baumanii* and Enterobacteriaceae. Currently, several structures of OXA-23 or its close correlates [26,27,28,29,30,31] are present in the PDB [32,33]. The OXA-23 protein, similarly to other members of class D β-lactameses, contains two domains, a mixed-α/β domain with two helices and a six-stranded β sheet, and an all-α domain, with the active site at the junction of the two domains [16,27]. An important breakthrough in understanding the catalytic mechanism of the OXA enzymes occurred when a conserved active site lysine has been identified to be carboxylated (after a reversible reaction with atmospheric carbon dioxide), leading to the proposal that this modified residue operates as general base for both the acylation and deacylation steps of the reaction [34]. Despite the importance of this enzyme, few studies concerning its dynamic characteristics are reported in the literature (see for example [27]). In this work, we report the results of a molecular dynamics analysis of OXA-23 in order to clarify some aspects of the functioning mechanism of this protein, which is knowledge necessary not only for a better understanding of its mechanism of action, but also for designing non-hydrolysable β-lactams or specific inhibitors of this class of enzymes.

## 2. Results

**Figure 2:**
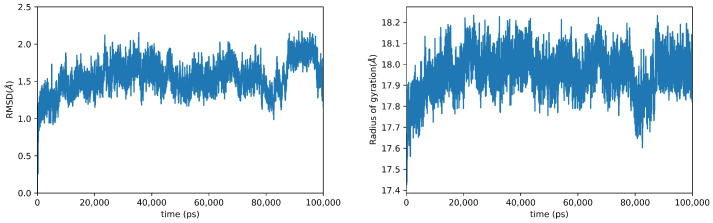
Analysis of the dynamics of OXA-23. The figure shows the time course of RMSD (**left panel**) and Rg (**right panel**) sampled at 10 ns. The RMSD was calculated considering only the backbone atoms and using as reference the crystallographic structure 4JF6.

In order to obtain information on the motions of OXA-23, we performed a 100 ns long molecular dynamics in an explicit solvent. The system obtained using the 4JF6 crystallographic structure after an initial relaxation phase (which is observed in all molecular dynamics experiments) reaches a stable steady state, which is maintained until the end of the simulation. The observation of the time course of the root-mean-square deviation (RMSD), a measure of the average distance between the protein atoms in a sampled conformation and the corresponding atoms of a reference conformation, confirms the stability of the simulation (see Figure 2, left panel). Although the simulation converged, the RMSD time course analysis clearly shows that the protein is quite dynamic and flexible, with significant conformational changes observed during the simulation. This same trend in the RMSD, which oscillates around 1.5 Å, is observed in a different simulation extended at 150 ns (see Supplementary Figure S1). This observation is further corroborated via the analysis of the radius of gyration (Rg), which, in the physical chemistry of polymers, is a measure of the dimensions of the molecule. Rg is reported in Figure 2, right panel. Although RMSD and Rg are not exactly linearly correlated, these analyses suggest that the protein undergoes significant conformational changes, moving from relatively compact conformations to more open ones. Although in part the variability of the RMSD and Rg can be attributed to the mobility of some loops on the surface of the protein (see below), a series of coherent motions of entire regions of the protein also contribute to the conformational variability. The OXA-23 protein folds into two noncontiguous domains, a mixed α/β domain with two α-helices and a six-stranded β-sheet, and an all-α domain, and the active site is located at the junction between the two domains. A measure of the reciprocal motion of these two domains is shown in Supplementary Figure S2, where the angle formed by residues Phe110, Kcx 82, and Met221 is reported (Kcx refers to the N-carboxylated lysine residue present in the active site of this class of enzymes). As can be appreciated from an inspection of the figure, the angle goes from values lower than 70° in closed conformations to values of 90° (and over) in open conformations. Similar values are also obtained for different choices of the atoms that define the angle. For example, by replacing the α-carbon of Phe110 with the equivalent of the Glu114 residue, the angle obtained takes on values ranging from 98° to 57°, with a linear relation between the two angles thus calculated.

**Figure 3:**
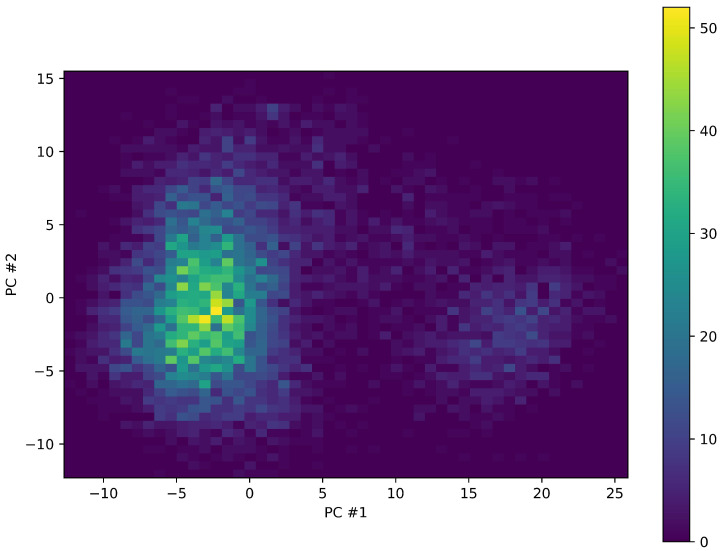
PCA analysis of the OXA-23 trajectory. The figure shows the distribution of the structures sampled during the simulation in the plane identified by the first two principal components. The plan has been divided into 50 bins for each component and the color represents the number of sampled structures belonging to each two dimensional bin (see the color scale alongside). Two regions are clearly observed, one on the left, more frequently visited, and one on the right in the figure, less frequently visited. In both regions, there is not a single maximum, but several peaks.

That the protein visits different conformations during the simulation is also demonstrated via principal component analysis (PCA). PCA is a widely used technique in exploratory data analysis and in multivariate statistics. It is used (also in this work) for dimensionality reduction, i.e., to reduce a high number of variables that describe a set of data (in our case the dynamics of the protein, described by three Cartesian coordinates for each atom and for each sampling time) to a smaller number of latent variables, simultaneously limiting the loss of information as much as possible [35,36]. PCA trajectory analysis of OXA-23 clearly shows that the protein visits two regions: one of these is visited much more frequently and is characterized by a complex structure. Figure 3 shows a two-dimensional histogram of the plane made up of the first two principal components. The most frequently visited basin shows a region with a more pronounced maximum (which should correspond to a region with lower Gibbs free energy under the simulation conditions), near which secondary peaks are observed. A more in-depth analysis shows that the minima visited in this region correspond to more or less open forms of the enzyme. The Supplementary Figure S3 shows an example of this type of open–closed–open transition. This shows that OXA-23 is a protein characterized by a remarkable conformational plasticity, probably important for its enzymatic activity. In addition to this, the protein also visits a second region in the principal component landscape, albeit at a lower frequency (Figure 3; see also Supplementary Figure S4). This too has a complex structure with some maxima in the distribution of the sampled structures (i.e., different minima from the point of view of free energy). The major differences between this region and the more populated one mentioned previously, however, concern the conformation of surface loops and amino-terminal residues, and therefore, they are presumably not very important for the purposes of the functionally important conformational plasticity we mentioned previously.

**Figure 4:**
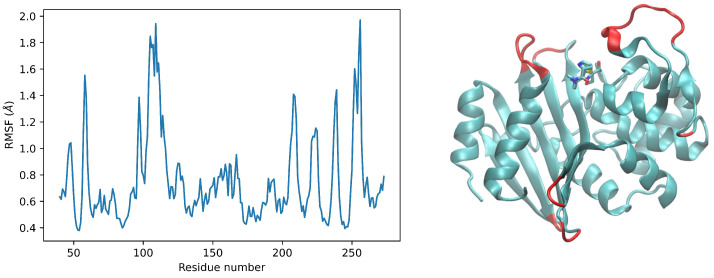
RMSF trajectory analysis of OXA-23. (**Left panel**) the graph shows the RMSF calculated for each residue as described in the Section 4. (**Right panel**) localization of the most mobile regions (in red) in the OXA-23 structure. To better highlight the position of the active site, the structure used is 4JF4, which corresponds to OXA-23 bound to meropenem [27].

To understand how this conformational plasticity is distributed in the OXA-23 structure, we calculated the root-mean-square-fluctuation (RMSF) of each residue. The RMSF of a structure is the time average of the RMSD and indicates which areas of the system are most mobile. The result of this analysis is shown in Figure 4. The RMSF clearly shows that not all of the protein undergoes significant fluctuations; indeed, most of the protein appears remarkably cool, with a very low RMSF. A particularly rigid region is the central one with respect to the amino acid sequence. The same Figure 4 shows the localization of protein regions characterized by high RMSF values. As expected, affected by a high RMSF value are mainly turn regions (see Figure 4). Of particular interest is an extended loop (ranging from residue 104 to 122) facing the active site of the protein. The localization of this high RMSF region, along with two other regions with significant mobility located on the opposite side of the active site, suggests that these movements may be important for the enzymatic activity of OXA-23. One possibility is that these highly flexible regions located above the active site, together with the coherent movements of the two domains and the rotameric configurations of some residues, may somehow be involved in the promiscuity of the enzyme, i.e., in its ability to efficiently accept different substrates. It is interesting to note that many conformations of the enzyme obtained via molecular dynamics are more similar to structures other than 4JF6, which was used as the starting structure, as shown in Supplementary Figures S5 and S6 (this analysis was carried out using the search function for structural similarity available in PDB).

**Figure 5:**
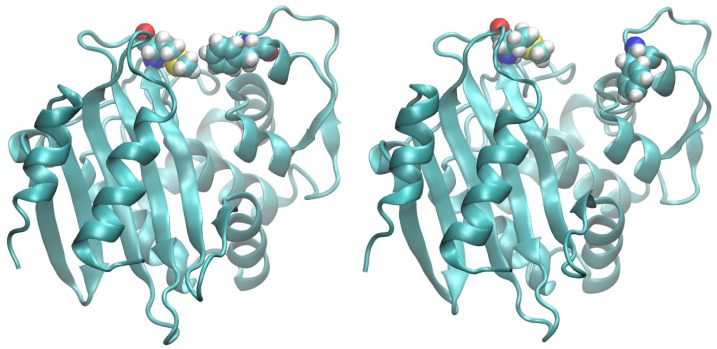
Hydrophobic bridge of OXA-23. The figure shows two conformations sampled during the simulation, one in which the distance between the Phe110 and Met221 residues is considerable, another in which the distance is very short. The two conformations have been superimposed; the two residues (Phe110 and Met221) are plotted as van deer Waals surfaces, using the following color code: carbon in light blue, oxygen in red, nitrogen in blue, sulfur in yellow and hydrogen in white.

Related to what has been described so far on the mobility of loops in the region around the active site is the presence of a hydrophobic bridge, which hinders the access to the active site. The structure of OXA-24/40 exhibits tunnel-like access to the active site due to a hydrophobic barrier formed by the side chains of Tyr112 and Met223 [37]. This arrangement has been suggested to be important in allowing entry into the active site of carbapenems because of their hydroxyethyl group, whereas access of β-lactam antibiotics with bulkier groups such as oxacillin and methicillin would be more difficult. Moreover this hydrophobic structure has been suggested to be important for the high affinity binding of carbapenem to OXA-24/40. In the structures of OXA-48 and OXA-58, this hydrophobic bridge is not visible either due to substitution of one of the two hydrophobic residues (Tyr213 in OXA-24/40 is in the position occupied by Thr213 in OXA-48) or due to a more open crystallographic structure (in the case of OXA-58) [24,38,39]. In the crystallographic structure of OXA-23 used in this work [27], the hydrophobic bridge is visible as in the case of OXA-24/40 (side chains of Phe110 and Met221). We monitored the distance between these two residuals throughout the simulation. What can be deduced is that the distance between these two residues (measured at the center of mass of each) varies greatly during the simulation, passing from values lower than 5 Å to values slightly higher than 25 Å. The switch is also quite fast and in the 100 ns of simulation, many transitions of this type can be observed. Figure 5 shows the configuration of the two residues at extreme values. This analysis therefore shows that both conformations observed crystallographically in carbapenemases are observable in the same enzyme (see also above). At least in the case of OXA-23, the enzyme can assume an open and closed conformation and therefore adapt to different substrates: in future works it would be interesting to see also if the other enzymes mentioned above can assume different conformations in dynamics and investigate how much these different conformations can contribute to the stability/binding selectivity of the various substrates.

**Figure 6:**
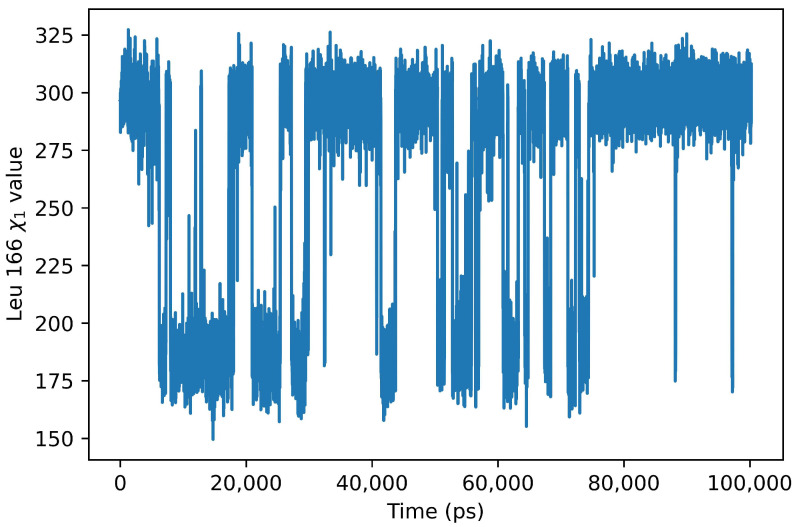
Rotamer conformations of leucine 166. The graph shows the value of the angle $\chi_{1}$ during the simulation. As can be seen, during the simulations, only two rotameric regions are allowed for the side chain of this amino acid.

A residue that has previously been identified as important for the activity of OXA-23 as a carbenepenase is Leu166. It has been suggested that this amino acid residue is important in regulating the access of water molecules to the active site. In the crystallographic structure of OXA-23, this residue assumes a conformation that allows for the formation of a channel that provides access for water molecules to the N-carboxylated Lys82. The simulation reported here shows that this amino acid residue is highly mobile, with conformational changes that could be important for the enzymatic activity and selectivity of this protein. To evaluate the mobility of this amino acid, we analyzed the rotameric conformations it is able to assume during the simulation. In particular, we measured the value of the angle $\chi_{1}$ of the side chain, and the result of this analysis is shown in Figure 6. As can be seen Leu166 presents two admissible values of the angle $\chi_{1}$ during the simulation, one between 175° and 200° and the other between 275° and 320°, with a prevalence of the time spent in the latter rotameric conformation. Inspection of the structures obtained during the simulation confirms the remarkable mobility of this amino acid residue. In fact, the side chain of Leu166 is found in two main differing positions: in one, it is placed in a sort of concavity, in which Trp165 participates. In another group of simulation snapshots, the Leu166 side chain is in a more extended conformation, distant from Trp165, which leans towards the Met221 side chain. This latter conformation of Leu166 can be observed particularly when the distance between the two residues, which form the hydrophobic bridge mentioned above, is greater.

Our analysis therefore confirms the peculiarity of this amino acid residue, regarding the mobility and the ability to regulate the access to the active site region of water molecules necessary for the deacylation reaction.

## 3. Discussion

Until recently considered a species of little importance from a clinical point of view, *A. baumanii* has reached vehemently impressive levels of danger in just a few years. *A. baumanii* currently represents a very serious problem from a clinical point of view considering the number of infections caused by this bacterium, particularly in intensive care units and in subjects already fragile due to concomitant pathologies, with impressive mortality data. It is no coincidence that it has been declared among the objectives for which it is of the utmost priority to find new therapies by the WHO. What makes *A. baumanii* extremely worrying is its ability to rapidly acquire mechanisms of resistance to antibiotics, so much so that finding strains of *A. baumanii* resistant to practically all antibiotics is no longer so unlikely in clinical practice.

One of the most worrying aspects of *A. baumanii* is its resistance to even last-resort antibiotics such as carbapenems. One of its major weapons are β-lactamases and, particularly, class D enzymes (see the Section 1). Despite their extreme clinical importance, relatively little work has been performed on these *A. baumanii* enzymes. In this work, we have analyzed the molecular dynamics of *A. baumanii* OXA-23: the analysis reported here highlights some of the peculiarities of this enzyme. Although in many respects similar to enzymes that do not have carbapenemase activity such as OXA-1, OXA-23 has some aspects that make it particular and which justify its peculiarity from the point of view of enzymatic action.

A rather interesting aspect that is highlighted by our analysis is that this enzyme is characterized by a remarkable structural plasticity. RMSD and Rg, as well as PCA, suggest that OXA-23 is a dynamic protein, with different conformations that are accessible at the temperature used for the simulation (303.15 K). Alongside the presence of mobile loops on the surface of the protein, the two domains show a significant reciprocal movement in the simulations we obtained. We highlight that this is probably a key factor from the point of view of OXA-23 activity. A plausible hypothesis is that the flexibility of the protein is the basis of its ability to act on a broad spectrum of β-lactams, including molecules belonging to the carbapenem class. As reported above, OXA-23 dynamically can assume conformations that are observed in different types of class D enzymes: in our analysis, we demonstrate that OXA-23 can assume both closed conformations, in which the hydrophobic tunnel is clearly visible, and open conformations, in which the residues participating in this tunnel (Phe110 and Met221) are considerably distant from each other. Furthermore, the conserved amino acid residue Leu166, which in enzymes not endowed with carbapenemase activity (e.g., OXA-1) appears rigid and not very mobile, in OXA-23, it can assume two different rotameric conformations. This results in the ability to control the access of water to the active site carboxylated lysine. Alongside this, it should be remembered the presence of a hydrophobic cage which appears to be peculiar to OXA enzymes endowed with carbapenemase activity consisting of Ile223 and Val227 and which participates together with Leu166 in controlling the access of the aqueous solvent to the region of the active site. This remarkable conformational flexibility is confirmed also via PCA, which shows that the protein visits two large clusters of conformations, with the presence of multiple structural minima nested into the larger two. These two large clusters differ from each other mainly due to different conformations of mobile regions on the protein surface, while the minima that are observed within these two clusters are due to open–closed conformational changes.

This conformational flexibility, in addition to being important for the functioning and substrate specificity of this enzyme, should also be considered for the rational design of new antibiotics capable of not being degraded by OXA-23 or for the design of molecules that selectively inhibit the activity of this enzyme. The presence of these different conformations, particularly in the neighborhood of the active site, with the possibility of observing closed structures and structures with the region of the active site more open and accessible, and the presence of residues capable of modulating the accessibility to the solvent should be considered in this type of analysis.

## 4. Materials and Methods

Molecular dynamics has been performed in NAMD [40] in a water box with 15 Å padding, as described [41,42,43] with modifications. Briefly, a CHARMM36m force field [44] was used, and parameterization of the protein was carried out by means of CHARMM-GUI [45,46,47]. Ionic strength and electroneutrality were obtained by adding potassium and chloride ions at a concentration of 150 mM. Periodic boundary conditions and the particle mesh Ewald (PME) method have been used; the time step was 2 fs. Systems underwent 10,000 conjugate gradient minimization steps followed by 125,000 equilibration steps in canonical ensemble conditions, with the protein fixed, after which 100 ns production runs began in the NpT ensemble (Langevin dynamics at 303.15 K and Nose–Hoover Langevin piston at 1.01325 bar). Structural analysis was conducted essentially as described in a VMD (version 1.9.3) environment [48,49,50,51].

Molecular dynamics analysis was performed in MDAnalysis [52,53] (version 2.1.0). In molecular dynamics analysis, RMSD is a measure of the average distance between the atoms, and, in order to minimize the RMSD, the rotational superposition of molecular structures should be performed before calculation [54]. Several algorithms have been proposed for finding the rotation that minimizes the squared distances between corresponding atoms in the structures. Methods based on the quaternion parameterization of rotation are fast, accurate, and robust, and are actually the methods of choice for the rotational superposition of structures obtained via molecular dynamics. In this work, we have used the method described in [55]: this quaternion-based characteristic polynomial (QCP) algorithm requires significantly less computation than the alternate methods. Moreover, it is fast and has been benchmarked on several computing platforms, including Linux. The crystallographic structure obtained from the PDB [32] entry 4JF6 [27] was used as a reference for RMSD analysis.

RMSF was calculated on the α-carbon atoms using the appropriate built-in function in the MDAnalysis suite v2.1.0 [52,53,55,56], implementing the algorithm described in [57]. The radius of gyration, a measure of the compactness of a protein structure, has been calculated using the built-in function in the MDAnalysis suite.

PCA is an orthogonal linear transformation in which a set of observations of possibly correlated variables map into a set of linearly uncorrelated new variables (the *principal components*). The first principal component has the largest variance and subsequent components are orthogonal with respect to the previous one. In this work, we used a PCA implementation based on the eigenvector decomposition of the correlation matrix [36,58]. Briefly, we assume that the data are arranged in a matrix such that each row represents a sampled conformation and each column represents a degree of freedom of the α-carbon atoms. After the centroid subtraction, the covariance matrix of the data set is obtained and the correlation matrix is calculated. This square symmetric matrix is diagonalized using standard numerical routines. The simulation matrix is projected onto the sorted eigenvector matrix to give the principal components. Numerical calculations were performed using Numpy (version 1.22.3) [59] in a Jupyter (Jupyter Notebook 6.4.11) environment [60]. Graphs were obtained in Matplotlib (version 3.5.2) [61].

## 5. Conclusions

In this study we have demonstrated how OXA-23 undergoes a series of conformational changes, some of which are probably correlated with the enzymatic activity of this protein, in the sense of creating the right environment necessary for the correct reaction as well as for substrate selectivity. Although part of the protein shows poor mobility, our analysis indicates that the region above the active site, thanks to the flexibility of the loops, coherent motions of the two protein domains, and the presence of conformers of some amino acid residues, undergoes open–closed conformational changes. We believe that taking into account what has been obtained from this molecular dynamics analysis is of fundamental importance for the search for ligands capable (hopefully) of inhibiting this protein of extreme clinical importance.

## Data Availability

In this work, public databases and freely available software resources have been used. All relevant data generated in this work are reported in the text. Additional data may be provided upon reasonable request to the authors.

## Supplementary Materials

The following supporting information can be downloaded at: https://www.mdpi.com/article/10.3390/ijms242417527/s1.

## References

[1] Fleming A. (1929). On the antibacterial action of cultures of a penicillium, with special reference to their use in the isolation of *B. influenzae*. Br. J. Exp. Pathol..

[2] Uddin T.M., Chakraborty A.J., Khusro A., Zidan B.R.M., Mitra S., Emran T.B., Dhama K., Ripon M.K.H., Gajdács M., Sahibzada M.U.K. (2021). Antibiotic resistance in microbes: History, mechanisms, therapeutic strategies and future prospects. J. Infect. Public Health.

[3] Aslam B., Wang W., Arshad M.I., Khurshid M., Muzammil S., Rasool M.H., Nisar M.A., Alvi R.F., Aslam M.A., Qamar M.U. (2018). Antibiotic resistance: A rundown of a global crisis. Infect. Drug Resist..

[4] De Oliveira D.M., Forde B.M., Kidd T.J., Harris P.N., Schembri M.A., Beatson S.A., Paterson D.L., Walker M.J. (2020). Antimicrobial resistance in ESKAPE pathogens. Clin. Microbiol. Rev..

[5] Mancuso G., Midiri A., Gerace E., Biondo C. (2021). Bacterial antibiotic resistance: The most critical pathogens. Pathogens.

[6] Romanelli F., Stolfa S., Morea A., Ronga L., Prete R.D., Chironna M., Santacroce L., Mosca A. (2021). Meropenem/vaborbactam activity in vitro: A new option for Klebsiella pneumoniae carbapenemase (KPC)-producing *Klebsiella pneumoniae* treatment. Future Microbiol..

[7] Arrigoni R., Ballini A., Topi S., Bottalico L., Jirillo E., Santacroce L. (2022). Antibiotic Resistance to *Mycobacterium tuberculosis* and Potential Use of Natural and Biological Products as Alternative Anti-Mycobacterial Agents. Antibiotics.

[8] Cazanave C., Manhart L.E., Bébéar C. (2012). Mycoplasma genitalium, an emerging sexually transmitted pathogen. Med. Mal. Infect..

[9] Hoffman S.J., Caleo G.M., Daulaire N., Elbe S., Matsoso P., Mossialos E., Rizvi Z., Røttingen J.A. (2015). Strategies for achieving global collective action on antimicrobial resistance. Bull. World Health Organ..

[10] Collier P., O’Neill L.J. (2018). Two years on: An update on achievement towards the recommendations of the antimicrobial resistance report. J. Appl. Microbiol..

[11] Davies S.C., Fowler T., Watson J., Livermore D.M., Walker D. (2013). Annual Report of the Chief Medical Officer: Infection and the rise of antimicrobial resistance. Lancet.

[12] Peleg A.Y., Seifert H., Paterson D.L. (2008). Acinetobacter baumannii: Emergence of a successful pathogen. Clin. Microbiol. Rev..

[13] Visca P., Seifert H., Towner K.J. (2011). Acinetobacter infection–an emerging threat to human health. IUBMB Life.

[14] Ramirez M.S., Bonomo R.A., Tolmasky M.E. (2020). Carbapenemases: Transforming *Acinetobacter baumannii* into a yet more dangerous menace. Biomolecules.

[15] World Health Organization (2017). Guidelines for the Prevention and Control of Carbapenem-Resistant Enterobacteriaceae, Acinetobacter baumannii and Pseudomonas aeruginosa in Health Care Facilities.

[16] Tooke C.L., Hinchliffe P., Bragginton E.C., Colenso C.K., Hirvonen V.H., Takebayashi Y., Spencer J. (2019). β-Lactamases and β-Lactamase Inhibitors in the 21st Century. J. Mol. Biol..

[17] Dever L.A., Dermody T.S. (1991). Mechanisms of bacterial resistance to antibiotics. Arch. Intern. Med..

[18] Bush K. (2018). Past and present perspectives on β-lactamases. Antimicrob. Agents Chemother..

[19] Abraham E.P., Chain E. (1940). An enzyme from bacteria able to destroy penicillin. Nature.

[20] Kirby W.M. (1944). Extraction of a highly potent penicillin inactivator from penicillin resistant staphylococci. Science.

[21] Beta Lactamase Data Base. http://www.bldb.eu/.

[22] Bush K., Jacoby G.A., Medeiros A.A. (1995). A functional classification scheme for beta-lactamases and its correlation with molecular structure. Antimicrob. Agents Chemother..

[23] Ambler R.P. (1980). The structure of β-lactamases. Philos. Trans. R. Soc. Lond. B Biol. Sci..

[24] Jeon J.H., Lee J.H., Lee J.J., Park K.S., Karim A.M., Lee C.R., Jeong B.C., Lee S.H. (2015). Structural basis for carbapenem-hydrolyzing mechanisms of carbapenemases conferring antibiotic resistance. Int. J. Mol. Sci..

[25] Yoon E.J., Jeong S.H. (2021). Class D β-lactamases. J. Antimicrob. Chemother..

[26] Kaitany K.C.J., Klinger N.V., June C.M., Ramey M.E., Bonomo R.A., Powers R.A., Leonard D.A. (2013). Structures of the class D carbapenemases OXA-23 and OXA-146: Mechanistic basis of activity against carbapenems, extended-spectrum cephalosporins, and aztreonam. Antimicrob. Agents Chemother..

[27] Smith C.A., Antunes N.T., Stewart N.K., Toth M., Kumarasiri M., Chang M., Mobashery S., Vakulenko S.B. (2013). Structural basis for carbapenemase activity of the OXA-23 β-lactamase from *Acinetobacter baumannii*. Chem. Biol..

[28] Mitchell J.M., Clasman J.R., June C.M., Kaitany K.C.J., LaFleur J.R., Taracila M.A., Klinger N.V., Bonomo R.A., Wymore T., Szarecka A. (2015). Structural basis of activity against aztreonam and extended spectrum cephalosporins for two carbapenem-hydrolyzing class D β-lactamases from *Acinetobacter baumannii*. Biochemistry.

[29] Harper T.M., June C.M., Taracila M.A., Bonomo R.A., Powers R.A., Leonard D.A. (2018). Multiple substitutions lead to increased loop flexibility and expanded specificity in *Acinetobacter baumannii* carbapenemase OXA-239. Biochem. J..

[30] Stewart N.K., Smith C.A., Antunes N.T., Toth M., Vakulenko S.B. (2019). Role of the hydrophobic bridge in the carbapenemase activity of class D β-lactamases. Antimicrob. Agents Chemother..

[31] Stewart N.K., Toth M., Alqurafi M.A., Chai W., Nguyen T.Q., Quan P., Lee M., Buynak J.D., Smith C.A., Vakulenko S.B. (2022). C6 hydroxymethyl-substituted carbapenem MA-1-206 inhibits the major *Acinetobacter baumannii* carbapenemase oxa-23 by impeding deacylation. Mbio.

[32] Berman H.M., Westbrook J., Feng Z., Gilliland G., Bhat T.N., Weissig H., Shindyalov I.N., Bourne P.E. (2000). The protein data bank. Nucleic Acids Res..

[33] Burley S.K., Bhikadiya C., Bi C., Bittrich S., Chen L., Crichlow G.V., Christie C.H., Dalenberg K., Di Costanzo L., Duarte J.M. (2021). RCSB Protein Data Bank: Powerful new tools for exploring 3D structures of biological macromolecules for basic and applied research and education in fundamental biology, biomedicine, biotechnology, bioengineering and energy sciences. Nucleic Acids Res..

[34] Golemi D., Maveyraud L., Vakulenko S., Samama J.P., Mobashery S. (2001). Critical involvement of a carbamylated lysine in catalytic function of class D β-lactamases. Proc. Natl. Acad. Sci. USA.

[35] Ringnér M. (2008). What is principal component analysis?. Nat. Biotechnol..

[36] Palese L.L. (2018). A random version of principal component analysis in data clustering. Comput. Biol. Chem..

[37] Santillana E., Beceiro A., Bou G., Romero A. (2007). Crystal structure of the carbapenemase OXA-24 reveals insights into the mechanism of carbapenem hydrolysis. Proc. Natl. Acad. Sci. USA.

[38] Docquier J.D., Calderone V., De Luca F., Benvenuti M., Giuliani F., Bellucci L., Tafi A., Nordmann P., Botta M., Rossolini G.M. (2009). Crystal structure of the OXA-48 β-lactamase reveals mechanistic diversity among class D carbapenemases. Chem. Biol..

[39] Smith C.A., Antunes N.T., Toth M., Vakulenko S.B. (2014). Crystal structure of carbapenemase OXA-58 from *Acinetobacter baumannii*. Antimicrob. Agents Chemother..

[40] Phillips J.C., Hardy D.J., Maia J.D., Stone J.E., Ribeiro J.V., Bernardi R.C., Buch R., Fiorin G., Hénin J., Jiang W. (2020). Scalable molecular dynamics on CPU and GPU architectures with NAMD. J. Chem. Phys..

[41] Bossis F., Palese L.L. (2013). Amyloid beta (1–42) in aqueous environments: Effects of ionic strength and E22Q (Dutch) mutation. Biochim. Biophys. Acta Proteins Proteom..

[42] Bossis F., De Grassi A., Palese L.L., Pierri C.L. (2014). Prediction of high-and low-affinity quinol-analogue-binding sites in the aa 3 and bo 3 terminal oxidases from *Bacillus subtilis* and *Escherichia coli*. Biochem. J..

[43] Sardanelli A.M., Isgrò C., Palese L.L. (2021). SARS-CoV-2 main protease active site ligands in the human metabolome. Molecules.

[44] Huang J., Rauscher S., Nawrocki G., Ran T., Feig M., De Groot B.L., Grubmüller H., MacKerell A.D. (2017). CHARMM36m: An improved force field for folded and intrinsically disordered proteins. Nat. Methods.

[45] Jo S., Kim T., Iyer V.G., Im W. (2008). CHARMM-GUI: A web-based graphical user interface for CHARMM. J. Comput. Chem..

[46] Brooks B.R., Brooks C.L., Mackerell A.D., Nilsson L., Petrella R.J., Roux B., Won Y., Archontis G., Bartels C., Boresch S. (2009). CHARMM: The biomolecular simulation program. J. Comput. Chem..

[47] Lee J., Cheng X., Swails J.M., Yeom M.S., Eastman P.K., Lemkul J.A., Wei S., Buckner J., Jeong J.C., Qi Y. (2016). CHARMM-GUI input generator for NAMD, GROMACS, AMBER, OpenMM, and CHARMM/OpenMM simulations using the CHARMM36 additive force field. J. Chem. Theory Comput..

[48] Bossis F., Palese L.L. (2011). Molecular dynamics in cytochrome c oxidase Mössbauer spectra deconvolution. Biochem. Biophys. Res. Commun..

[49] Palese L.L. (2015). Correlation analysis of Trp-cage dynamics in folded and unfolded states. J. Phys. Chem. B.

[50] Palese L.L. (2015). Random matrix theory in molecular dynamics analysis. Biophys. Chem..

[51] Humphrey W., Dalke A., Schulten K. (1996). VMD: Visual molecular dynamics. J. Mol. Graph..

[52] Michaud-Agrawal N., Denning E.J., Woolf T.B., Beckstein O. (2011). MDAnalysis: A toolkit for the analysis of molecular dynamics simulations. J. Comput. Chem..

[53] Gowers R.J., Linke M., Barnoud J., Reddy T.J., Melo M.N., Seyler S.L., Domanski J., Dotson D.L., Buchoux S., Kenney I.M. MDAnalysis: A Python package for the rapid analysis of molecular dynamics simulations. Proceedings of the 15th Python IN Science Conferences (SCIPY 2016).

[54] Flower D. (1999). Rotational superposition: A review of methods. J. Mol. Graph. Model..

[55] Theobald D.L. (2005). Rapid calculation of RMSDs using a quaternion-based characteristic polynomial. Acta Crystallogr. A.

[56] Liu P., Agrafiotis D.K., Theobald D.L. (2010). Fast determination of the optimal rotational matrix for macromolecular superpositions. J. Comput. Chem..

[57] Welford B. (1962). Note on a method for calculating corrected sums of squares and products. Technometrics.

[58] Raschka S. (2015). Python Machine Learning.

[59] Harris C.R., Millman K.J., van der Walt S.J., Gommers R., Virtanen P., Cournapeau D., Wieser E., Taylor J., Berg S., Smith N.J. (2020). Array programming with NumPy. Nature.

[60] Jupyter. https://jupyter.org/.

[61] Hunter J.D. (2007). Matplotlib: A 2D graphics environment. Comput. Sci. Eng..

