# Supplementary material for: The Dynamics of OXA-23 β-Lactamase from Acinetobacter baumannii
Source: Int J Mol Sci. 2023 Dec 15;24(24):17527. doi: 10.3390/ijms242417527 (PMC10743560; doi:10.3390/ijms242417527)
Supplement: Supplementary file 1 [file ijms-24-17527-s001.zip › ijms-2736995-supplementary.pdf]

The Dynamics of OXA-23 β-Lactamase from *Acinetobacter baumannii*

Roberto Arrigoni, Andrea Ballini, Luigi Santacroce and Luigi Leonardo Palese

Supplementary Figures

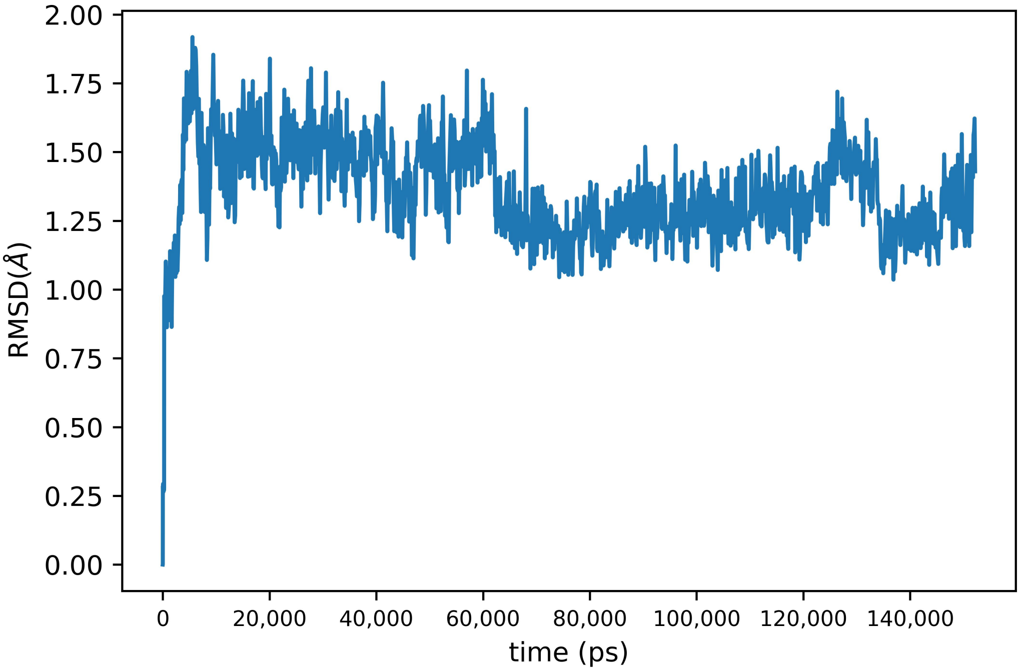

Figure S1. ***RMSD analysis of a 150 ns simulation.*** The RMSD was calculated considering only the backbone atoms and using as reference the crystallographic structure 4JF6. Note that in this case the sampling rate was 100 ps.

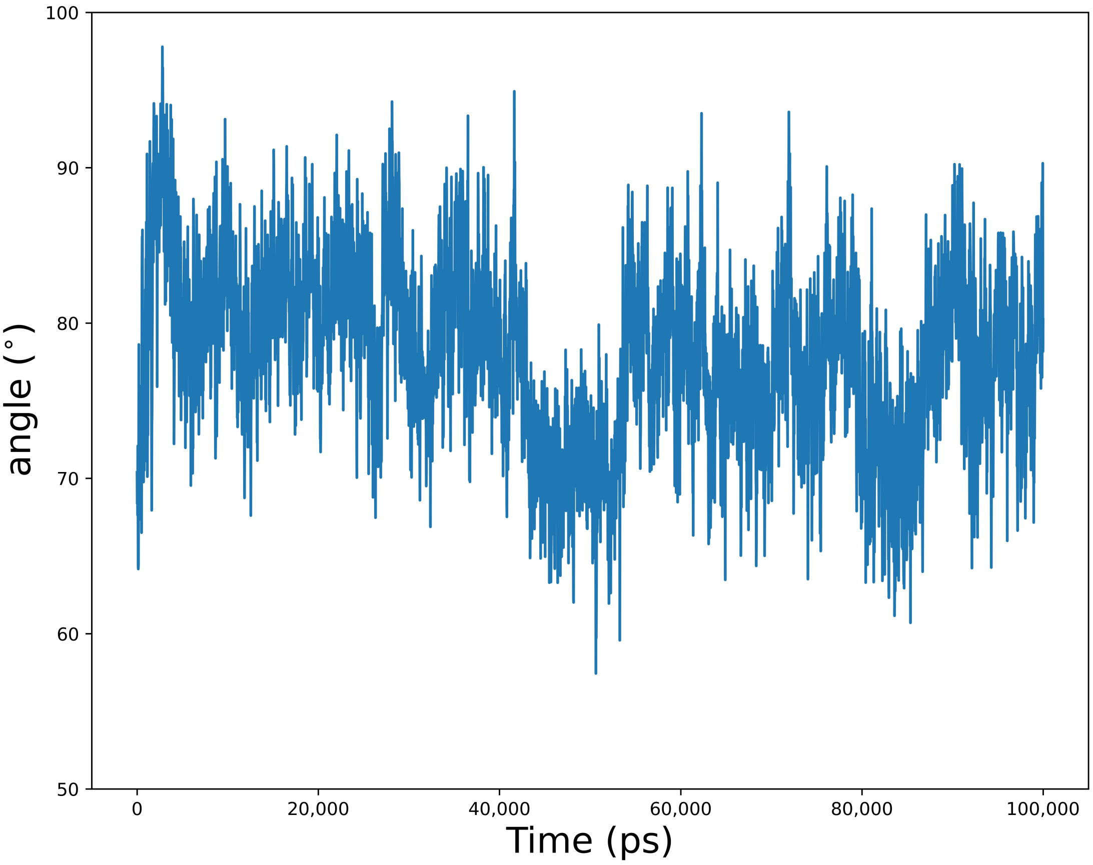

Figure S2 . ***Angle between the two domains of OXA-23.*** The figure shows the angle between the α-C of Phe 110, the ε-C of Kcx 82, and the α-C of Met 221. The figure refers to the 100 ns simulation discussed in the main text, sampled every 20 ps. The residue indicated as Kcx refers to the N-carboxylated lysine present in the active site of class D β-lactamases. The angle goes from values between 60° and 70° in the closed conformations of the enzyme, to values of 90° and further in open conformations.

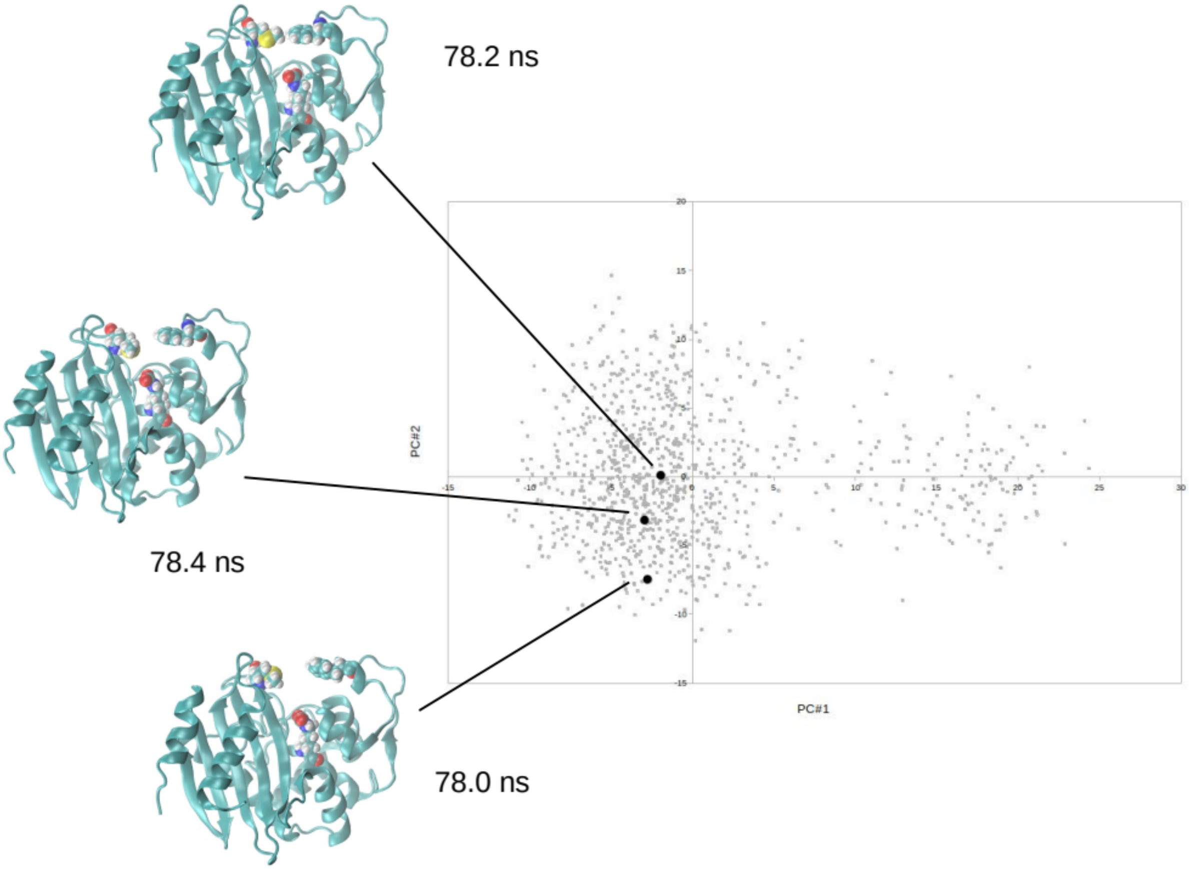

Figure S3. ***PCA of 100 ns simulation.*** The figure shows the PCA of the simulation discussed in the main text. The points shown as gray symbols are the conformations sampled every 100 ps. The black circles correspond to the structures shown alongside (sampled at 78.0, 78.2 and 78.4 ns). In the sequence shown we observe the transition from a more open conformation to a closed one and again to an open one. Residues Phe110, Met221, and Kcx 82 are reported as van der Waals radii (color code as in Figure 5 of the main text).

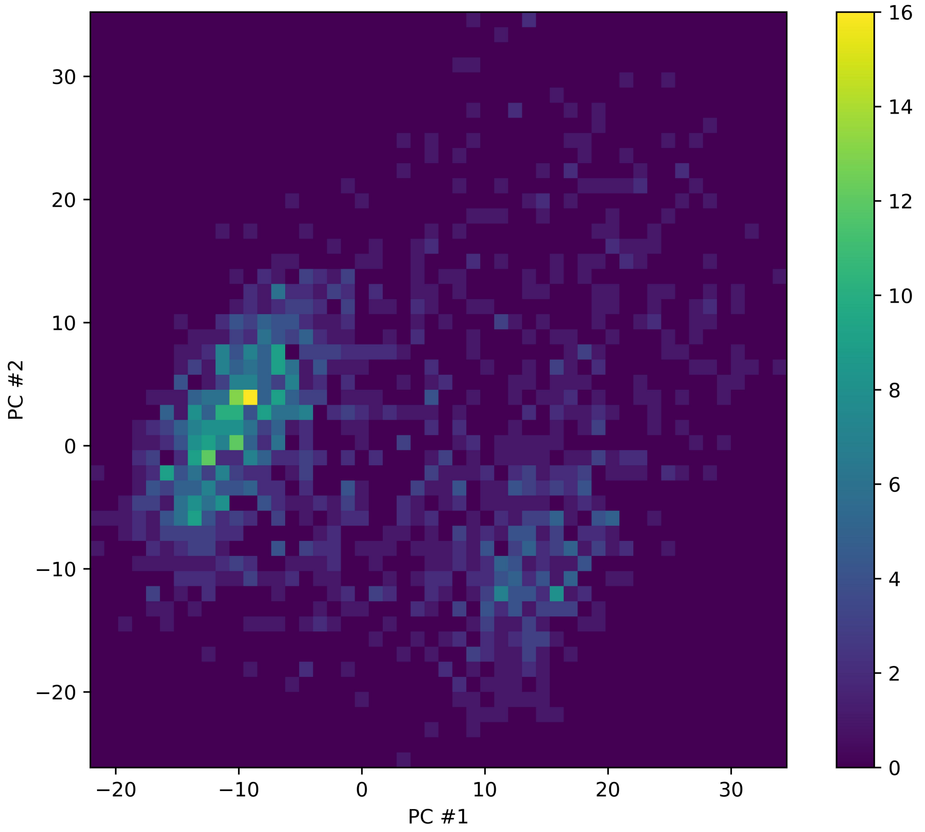

Figure S4. ***PCA of 150 ns simulation.*** The figure shows the distribution of the structures sampled during the simulation in the plane identified by the first two principal components. The plan has been divided into 50 bins for each component and the color represents the number of sampled structures belonging to each two dimensional bin (see the color scale alongside). Two regions are clearly observed, one on the left more frequently visited and one on the right in the figure, less frequently visited.

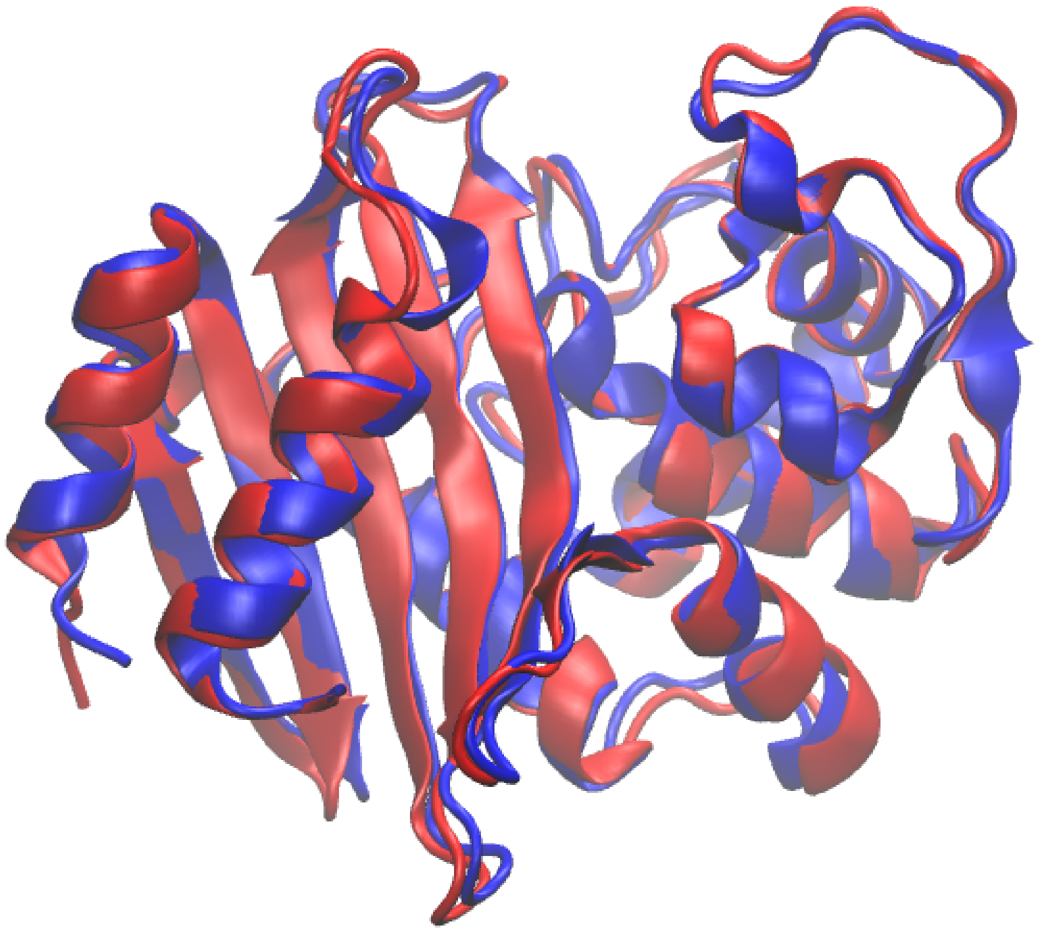

Figure S5. ***Superposition of a structure obtained through molecular dynamics with a crystallographic structure.*** The figure shows the overlap between an open conformation of the enzyme obtained via molecular dynamics (in red; frame sampled at 78.2 ns in the 100 ns simulation discussed in the text) and the 6N6X crystallographic structure (in blue; this PDB entry is described in ref. 30 of the main text).

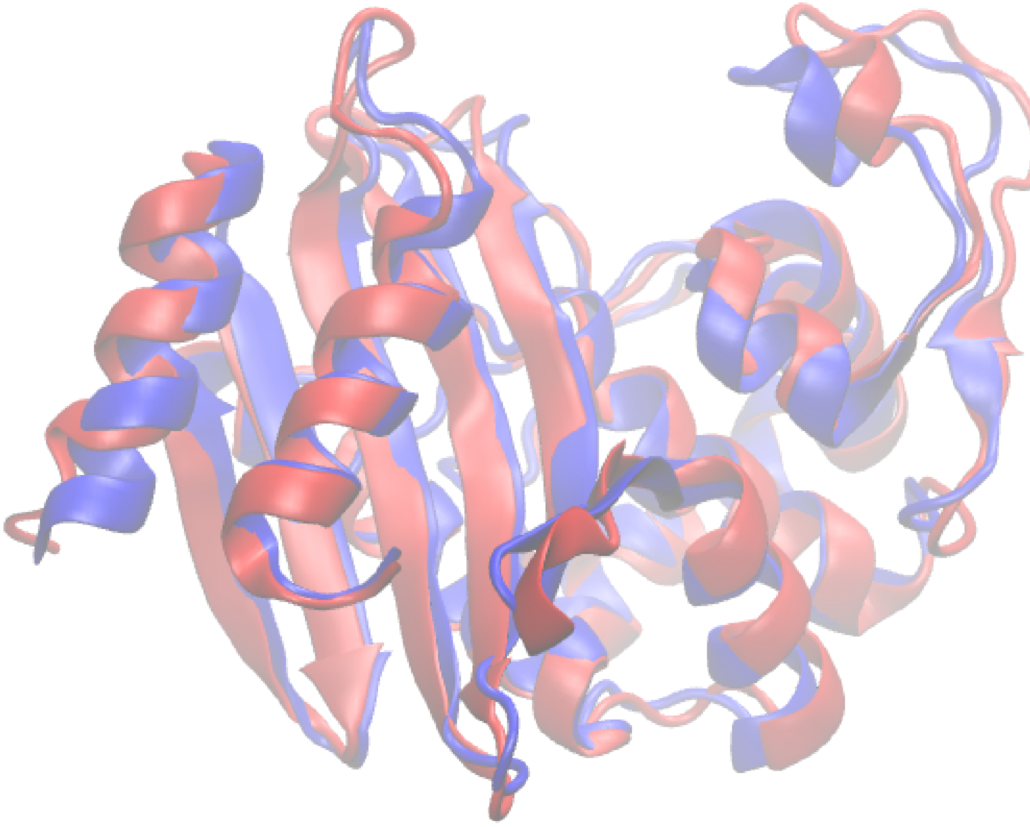

Figure S6. ***Superposition of a structure obtained through molecular dynamics with a crystallographic structure.*** The figure shows the overlap between an open conformation of the enzyme obtained via molecular dynamics (in red; frame sampled at 38.3 ns in the 100 ns simulation discussed in the text) and the 5KZH crystallographic structure (in blue).
